# Thermotolerant class A acid phosphatase active across broad pH range and diverse substrates

**DOI:** 10.1002/pro.70244

**Published:** 2025-08-15

**Authors:** Maria‐Isabel Recio, José A. Gavira, Jesús de La Torre, Mario Cano‐Muñoz, Sergio Martínez‐Rodriguez, Abdelali Daddaoua, Estrella Duque, Juan L. Ramos

**Affiliations:** ^1^ Estación Experimental del Zaidín Consejo Superior de Investigaciones Científicas Granada Spain; ^2^ Consejo Superior de Investigaciones Científicas Instituto Andaluz de Ciencias de la Tierra Armilla Spain; ^3^ Departamento de Bioquímica y Biología Molecular III e Inmunología Universidad de Granada Granada Spain; ^4^ Departamento de Bioquímica y Biología Molecular II Facultad de Farmacia, University of Granada Granada Spain

**Keywords:** bacteria, bacterial acid phosphatase, biomineralization, *Pseudomonas*, structure–function, substrate spectrum

## Abstract

M2‐32 is a non‐specific acid phosphatase with a rare ability to function across a broad pH range (3.5–8.5). Analysis using SWISS‐PROT Prf Profiles classifies it as a class A acid phosphatase (*Z*‐score: 78.97), sharing 50%–60% sequence similarity with enzymes such as PhoC and PhoN. For detailed characterization, the gene encoding M2‐32 was cloned into the pET28(b) vector, overexpressed in *Escherichia coli* BL21 (DE3), and subsequently purified. Although the monomeric form of M2‐32 has a molecular weight of ~28 kDa, size exclusion chromatography, dynamic light scattering, and sedimentation studies revealed a dimeric form in solution. Enzymatic assays using *p*‐nitrophenyl phosphate, 4‐methylumbelliferyl phosphate, 3′‐and 5′‐adenosine monophosphate demonstrated robust activity over a pH range of 4.0–8.0 at both 30 and 50°C. Differential scanning fluorimetry indicated an unfolding temperature close to 47°C; however, the enzyme refolded after heat denaturation at 80°C. We have determined the x‐ray crystal structure of M2‐32 by molecular replacement using an AlphaFold2‐guided truncated model, achieving a resolution of 2.2 Å. The protein crystallized as a dimer‐of‐dimers. Each monomer (residues 38–274) adopts an all‐alpha‐helical fold composed of 14 helices and two disulfide bonds. Docking studies with adenosine monophosphates, combined with site‐directed mutagenesis, identified His174, Arg207, His213, Asp217 as critical catalytic residues, and Tyr136 and Ser172 probably involved in substrate recognition. Mutations at these positions resulted in over 90% loss of enzymatic activity, highlighting their functional significance.

## 1 INTRODUCTION

Phosphorus is an essential macronutrient involved in the synthesis of critical macromolecules such as DNA, RNA, and phospholipids. In addition, various phosphorylated metabolites serve as relevant cofactors, metabolic intermediates, and signaling molecules in all biological kingdoms (Vance, 2011).

Monophosphoesterases, commonly known as phosphatases, release inorganic phosphate by dephosphorylating phosphodiester or phosphoanhydride bonds in organophosphorus compounds (Vance, 2011). These enzymes play a critical role in the mineralization of these chemicals, contributing significantly to the phosphorus cycle (Duhamiel, 2025; Eivazi & Tabatabai, 1977). Phosphatases are broadly classified as either alkaline or acid, based on their optimal pH range (Duhamiel, 2025). Beyond this functional classification, numerous families have been identified in eukaryotic and prokaryotic organisms based on sequence homology (Gandhi & Chandra, 2012; Neal et al., 2018; Udaondo et al., 2020). While alkaline phosphatases have been extensively studied in natural environments (Duhamiel, 2025), particularly due to the prevalent alkaline pH in aquatic ecosystems and because they seem to be more abundant (Duhamiel, 2025; McComb et al., 2013; Ragot et al., 2015, 2017), the information and importance of acid phosphatases in the environment remain underexplored. Nevertheless, Margalef et al. (2017) have emphasized their ecological relevance, particularly in soil. Recent research (Janes‐Bassett et al., 2022) confirmed their role in P cycling, especially in agricultural soil under low‐ or no‐tillage practices.

Generally, bacterial acid phosphatases are non‐specific enzymes with broad substrate specificity. They can be secreted in a soluble form or be retained as membrane‐bound proteins that scavenge organophosphoesters that are not transported into the cytoplasm (Lidbury et al., 2022). Acid phosphatases can also function intracellularly, playing roles in central metabolism and signaling processes (Lidbury et al., 2022). Initial classification of acid phosphatases was based on short sequence motifs (i.e., PROSITE, PRINTS, TIGRFAM, etc.), though these methods often produced discrepancies in the assignation of acid phosphatase to different classes. To address this, we and others have applied SWISS‐PROT profile‐based classification strategies, both in eukaryotic and prokaryotic contexts (Udaondo et al., 2020). These profiles offer great precision in identifying acid phosphatases and facilitate the accurate annotation of acid phosphatases deposited in genomic and metagenomic datasets (Udaondo et al., 2020). Based on sequence analysis, three distinct classes of prokaryotic acid phosphatases (referred to classes A, B, and C) were identified (Gandhi & Chandra, 2012; Neal et al., 2018; Udaondo et al., 2020). Classes A and C acid phosphatases are widely distributed among microbes and environmental samples, while class B acid phosphatases appear commonly associated with pathogens (Gandhi & Chandra, 2012).

Udaondo et al. (2020) applied sequence‐based profiling to scan genomes and metagenomes, successfully identifying numerous formerly unknown acid phosphatases belonging to the three classes. In vitro synthesis of genes encoding these proteins, followed by enzymatic assays, confirmed that these bioinformatically identified proteins were indeed acid phosphatases (Udaondo et al., 2020). Another effective approach for identifying phosphatases involves the screening of functional metagenomic libraries using indicator substrates such as 5‐bromo‐4‐chloro‐3‐indolyl phosphate (BCIP), which produces blue indigo stained colonies upon hydrolysis (Castillo‐Villamizar et al., 2019). Both sequence‐ and functional‐based approaches are valuable for discovering novel acid phosphatases with the aim of exploring their potential biotechnological applications, particularly those that are robust enzymes that can operate under harsh environmental conditions, such as high temperatures and a wide pH range. With this in mind, we hypothesized that screening fosmid metagenomic libraries expressed in *Escherichia coli* would yield phosphatases exhibiting unusual properties, including operation across broad pH ranges and thermotolerance. In this study, we have identified and characterized M2‐32, a thermostable class A acid phosphatase capable of refolding after denaturation at temperatures of 80°C, which exhibits robust activity in a pH range between 4.0 and 8.5, making it a promising candidate for environmental and industrial applications.

## 2 RESULTS

### 2.1 Identification of a non‐specific acid phosphatase recovered from a metagenomic library

Several metagenomic libraries derived from environmental samples (ruminal fluids, soils, and fresh water sources) were available in our group (Duque et al., 2018; Udaondo et al., 2020). Functional screening identified in a soil metagenomic library a clone harboring the fosmid FOS M2‐32, that encoded a phosphatase capable of hydrolyzing *p*‐nitrophenyl phosphate (pNPP) over a wide range of pH, that is, from 4.0 to 8.5. Sequencing of the fosmid revealed a putative acid phosphatase made up of 274 amino acids with an estimated molecular weight of 28.8 kDa. To classify this enzyme within one of the three recognized bacterial acid phosphatase classes, we used the SWISS‐PROT Prf profiles developed by Udaondo et al. (2020). The M2‐32 phosphatase showed no significant matches to class B or class C profiles (*Z*‐score ≤8.5). In contrast, it displayed a high *Z*‐score of 78.97 against the class A profile, strongly supporting that this enzyme belongs to class A acid phosphatases. Further comparisons of M2‐32 against UniProt‐K and NCBI database indicated that M2‐32 belongs to a group of acid phosphatases that included enzymes such as PhoC from *Zymomonas mobilis* and *Morganella morganii* (Pond et al., 1989; Thaller et al., 1994), PhoN from *Salmonella typhimurium* and *Morganella morganii* (Kasahara et al., 1991; Uchiya et al., 1996), and 1D2T from *Escherichia blattae* (Ishikawa et al., 2000, 2002). Sequence alignment of M2‐32 with these enzymes revealed only 50%–60% similarity, warranting detailed characterization of M2‐32 (Figure S1).

### 2.2 Molecular mass and oligomeric state of M2‐32

We cloned the DNA encoding M2‐32 into the expression vector pET28(b) to generate pET28::M2‐32 (Table S1). Overexpression of the gene in *E. coli* followed by purification of the protein to homogeneity confirmed a monomeric weight of approximately 28 kDa, consistent with the expected size, which is similar to that of proteins of the family (e.g., from 25 kDa for *M. morganii* [Thaller et al., 1994] to 30 kDa for *E. blattae* [Ishikawa et al., 2000]).

**FIGURE 1:**
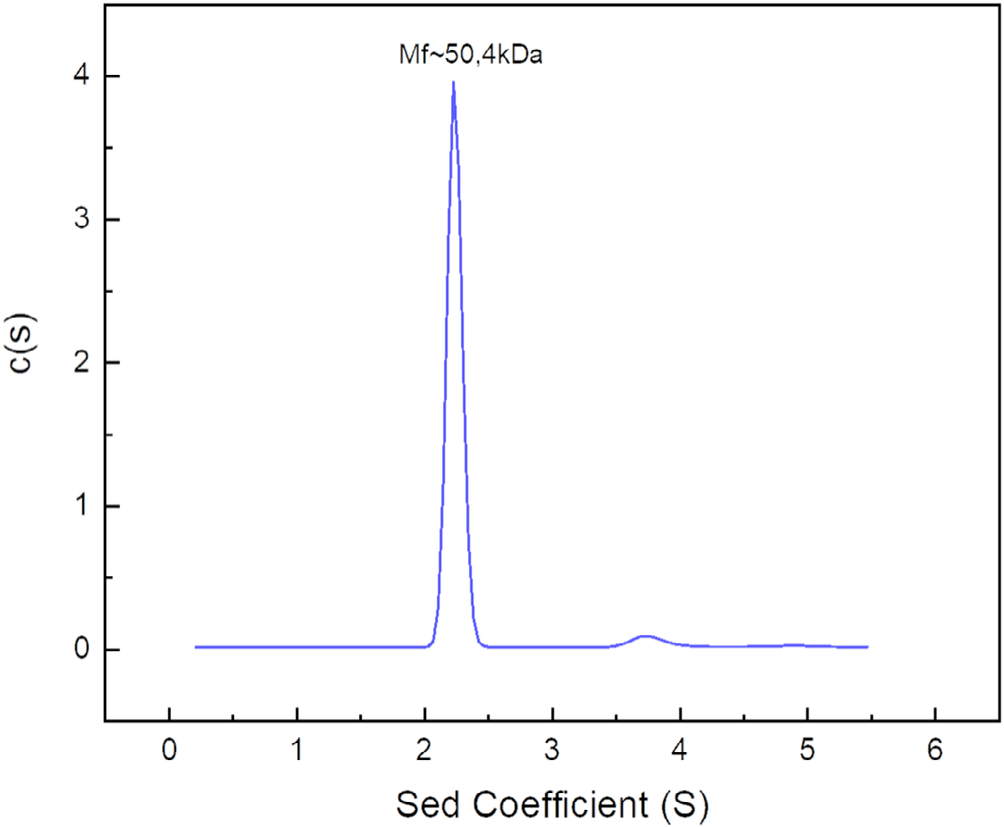
Sedimentation velocity analytical ultracentrifugation of M2‐32. The assays were conducted at 20°C, using an AnTi50 rotor, under the specific conditions described in Section 5.

To get further insights on the shape and quaternary structure of M2‐32, dynamic light scattering and sedimentation rate analysis were undertaken. The results revealed an *f*/*f*0 ratio of 1.38, indicating a relatively globular protein in shape. The sedimentation coefficient was 2.250, with over 97% of the protein present as a single species in the gradient (Figure 1 and Table S2). When corrected to standard conditions (the density and viscosity of water at 20°C), the sedimentation coefficient (*S*_20,w_) was calculated to be 3.632 (Table S2), corresponding to a molecular mass of ~50.4 kDa, suggesting that M2‐32 was a dimer in solution. To further confirm the molecular mass protein and dimeric state of the protein in solution, we carried out size exclusion chromatography using a HiPrep 26/60 Sephacryl S 500R column along with a set of globular proteins as molecular weight markers (see Section 5 for details). The M2‐32 protein eluted from the column with an apparent molecular mass of about 57.6 kDa, consistent with the results obtained from ultracentrifugation assays, and supporting the hypothesis that the protein is a dimer in solution (Figure S2).

### 2.3 M2‐32 is active over a broad range of temperatures and pHs

**FIGURE 2:**
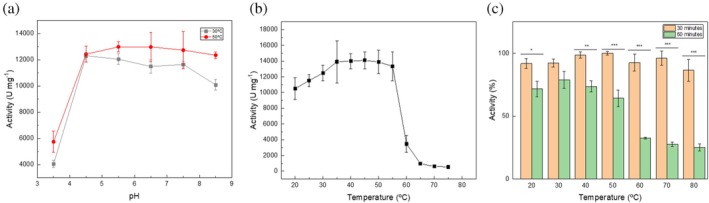
(a) Activity profile of M2‐32 as a function of pH at temperature of 30°C (gray line) and 50°C (red line). The values reported are the average of at least three independent assays in triplicate. (b) Activity profile of M2‐32 as a function of temperature. The pH of the incubation buffer was set at 5.5 and the values reported are the average of at least three independent experiments in triplicate. (c) Activity profile of M2‐32 after 30 min (orange bar) and 1 h (green bar) incubation at the indicated temperature, thereafter activity assays were carried out at 25°C. Statistical analysis was carried out using ANOVA; *p < 0.05; **p < 0.01; ***p < 0.001.

M2‐32 enzymatic activity was routinely assessed using pNPP as the substrate (see Section 5). With this substrate, M2‐32 exhibited activity in a broad pH range (between 3.5 and 8.5), with optimal activity between pH 4.0 and 8.0 at both 30 and 50°C (Figure 2a). At pH 3.5, the activity decreased by 50%, and at pH 8.5, the activity only slightly decreased (Figure 2a). To further investigate the stability of the protein under extreme pH conditions, the purified enzyme was dialyzed against the same buffer adjusted to pH 3.5 and 8.5, respectively. Enzymatic activity was then measured over time under standard conditions, and we found that regardless of the pH of the dialysis buffer, the enzyme maintained maximal activity. In parallel, temperature activity profiles were evaluated. The enzyme showed high activity (≥10^4^ U/mg protein) at temperatures between 25 and 55°C (Figure 2b), with maximal activity at 35–50°C. The activity declined sharply above 55°C but retained about 25% of maximum activity at 60°C.

We carried out thermal stability assays (Figure 2c) by incubating the enzyme for 30 or 60 min at temperatures from 20 to 80°C. After incubation, the samples were cooled at room temperature and the activity was assayed at 30°C. The results indicated that M2‐32 retained near 100% activity after a 30‐min incubation at temperatures up to 80°C. However, a 60‐min incubation at 60–80°C (Figure 2c) resulted in a 70%–80% loss of activity, which was statistically significant (*p* value <0.001). In contrast, only a moderate, yet statistically significant *p* value (<0.05) decrease in activity was observed following incubation at 20–50°C for 60 min.

**FIGURE 3:**
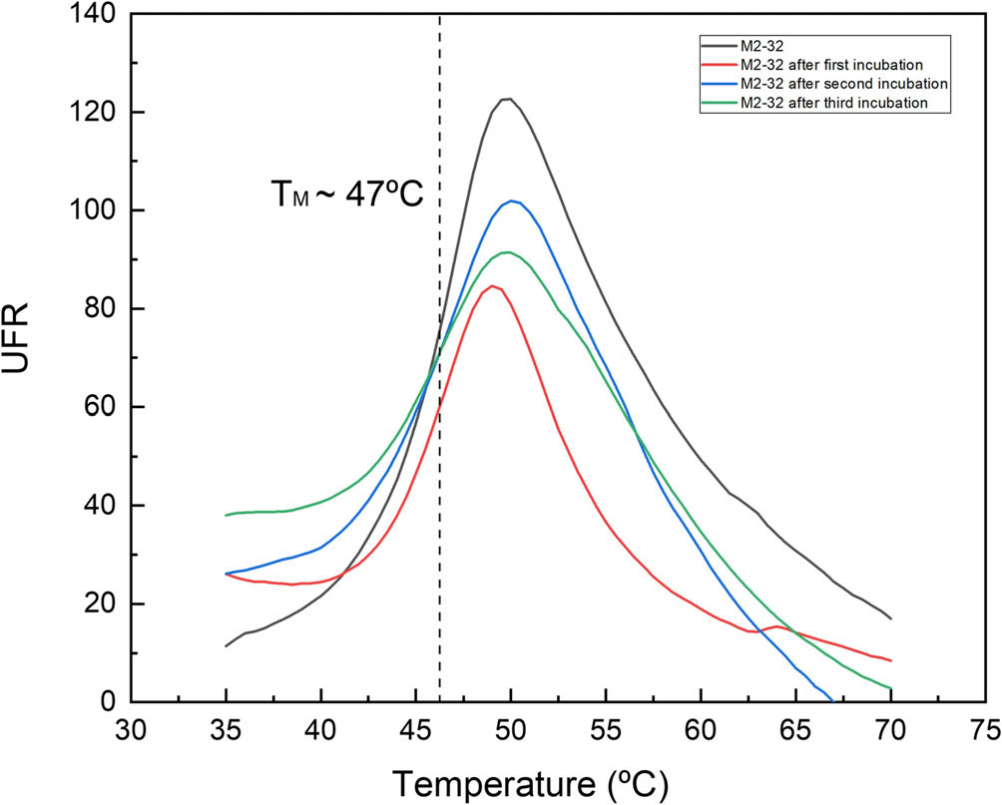
Thermal unfolding behavior of M2‐32. The protein was purified to homogeneity as described in Section 5. The protein concentration was determined and the M2‐32 protein was diluted in HAM buffer (pH 7.0) to a final concentration of 5 μM. Then the effect of the incubation temperature on the thermal stability of M2‐32 was assessed by measuring Relative Fluorescence Units (UFR) (black line). The unfolding temperature was determined using the Bio‐Rad iQS software. In a separate set of assays, the protein was first heated to 80°C, cooled at room temperature for 1 h and then thermal stability determined (red line). The procedure was repeated twice more (blue and green lines). The *T*_m_ was determined in each round after unfolding and refolding.

We also determined the thermal behavior of M2‐32 using differential scanning fluorimetry (DSF), which revealed the unfolding temperature point (*T*_m_). The assay, which was done, as described in Section 5, indicated that the *T*_m_ is of approximately 47°C (Figure 3). Shimanovich and Hartl (Shimanovich & Hartl, 2024) described the complex landscape of protein structures and unveiled that the complexity is the result of intramolecular interactions that drive folding, mainly non‐covalent and energetically weak interactions. Since M2‐32 is thermoresistant and works at high temperatures, we tested if the unfolded M2‐32 protein after incubation at high temperature spontaneously refolded. We investigated its refolding capacity after incubation for 60 min at 80°C, the samples were then cooled to 25°C and left for 2 h before the *T*_m_ was determined again. We found that M2‐32 was able to refold up to at least four consecutive times without altering its *T*_m_, making it a promising candidate for industrial applications requiring prolonged exposure to extreme temperatures (Figure 3).

### 2.4 Substrate profile

Phosphatases are generally non‐specific enzymes capable of hydrolyzing various substrates (Makde et al., 2007). To characterize the substrate profile of M2‐32, we used a battery of organophosphorus compounds and measured phosphate release at four different pHs (Table S3). Notably, M2‐32 hydrolyzed pNPP, adenosine 3′‐monophosphate (3′‐AMP), adenosine 5′‐monophosphate (5′‐AMP), and 4‐methylumbellifryl phosphate in the range of pH between 4 and 8.5; other compounds such as uridine 5′‐monophosphate, uridine 3′‐monophosphate, *o*‐phospho *L*‐serine, glucose‐6‐phosphate, or carbamyl phosphate were not dephosphorylated by the enzyme. This set of results indicates that M2‐32 exhibits a substrate profile comparable to those of other characterized bacterial acid phosphatases (Kasahara et al., 1991; Makde et al., 2007; Pond et al., 1989).

We also determined the kinetic parameters of M2‐32 using two artificial substrates (pNPP and 4‐methylumbelliferyl phosphate [4‐MUP]) and two natural substrates 3′‐AMP and 5′‐AMP (Table 1). For pNPP, the Michaelis constant (*K*_m_) was in the range of 0.34–0.49 mM at 25°C (Table 1). *V*_max_ with pNPP did not change significantly at different pHs (1.62–3.16 × 10^−7^ M/s) highlighting the robustness of M2‐32 (Table 1). Compared to other class A acid phosphatases such as those of *S. typhimurium* (*K*_m_ = 0.12 mM) and *P. intermedia* (*K*_m_ = 0.24 mM), the *K*_m_ of M2‐32 was slightly higher, suggesting lower affinity than other class A acid phosphatases (Kasahara et al., 1991; Makde et al., 2007; Pond et al., 1989). With the second artificial substrate, 4‐MUP the affinity and *V*_max_ were higher than with pNPP, with *K*_m_ values in the range of 0.05 mM and *V*_max_ in the range of 7 × 10^−5^ M/s. When the kinetic behavior of M2‐32 was tested with 5′‐AMP and 3′‐AMP, two natural substrates, at pH 5.5, the *K*_m_ was 0.1–0.26 mM, and *V*_max_ with the nucleotides was at least 100‐fold higher than with pNPP (Table 1). Catalytic efficiency with the two natural substrates was significantly higher than with the artificial pNPP substrate (Table 1). To evaluate the differences in each constant with the various substrates based on the assay pH, ANOVA statistical analysis was carried out. No significant differences were observed (*p* value <0.05), confirming the robustness of the M2‐32 protein.

**TABLE 1:** Kinetic parameters of M2‐32 and M2‐32 Y136A at different pH values.

Protein	Compound	pH	*K*_m_ (mM)	*V*_max_ (M/s)	*K*_cat_ (s^−1^)
M2‐32	pNPP	4	0.44 ± 0.10	2.27E−07 ± 7.4E−08	0.22 ± 0.19
		5.5	0.48 ± 0.11	3.16E−07 ± 6.44E−08	0.32 ± 0.06
		7	0.49 ± 0.12	2.98E−07 ± 5.6E−08	0.30 ± 0.05
		8.5	0.34 ± 0.10	1.6E−07 ± 9.1E−08	0.17 ± 0.09
	5′‐AMP	4	0.27 ± 0.1	5.50E−05 ± 1.83E−05	55 ± 10
		5.5	0.10 ± 0.03	7.63E−05 ± 1.74E−05	76 ± 17
		7	0.271 ± 0.1	6.48E−05 ± 1.98E−05	65 ± 19
		8.5	0.2 ± 0.1	1.4E−05 ± 3.36E−06	13 ± 3
	3′‐AMP	4	0.24 ± 0.04	1.06E−04 ± 1.67E−05	106 ± 16
		5.5	0.25 ± 0.01	1.10E−04 ± 6.53E−06	110 ± 6
		7	0.26 ± 0.03	1.08E−04 ± 8.53E−06	107 ± 8
		8.5	0.21 ± 0.02	1.23E−04 ± 8.49E−05	103 ± 35
	4‐MUP	4	0.05 ± 0.002	6.83E−05 ± 1.92E−06	68 ± 2
		5.5	0.06 ± 0.007	7.36E−05 ± 6.67E−05	73 ± 7
		7	0.05 ± 0.002	7.02E−05 ± 2.03E−06	70 ± 2
		8.5	0.05 ± 0.008	7.83E−05 ± 8.53E−06	78 ± 8
Y136A	pNPP	5.5	0.257 ± 0.045	5.43E−08 ± 1.10E−08	0.05 ± 0.01

Abbreviations: pNPP, *p*‐nitrophenyl phosphate; 3′‐AMP, adenosine 3′‐monophosphate; 5′‐AMP, adenosine 5′‐monophosphate; 4‐MUP, 4‐methylumbelliferyl phosphate.

Isothermal titration calorimetry assays suggested that the hydrolysis reactions at pH 5.5 with all substrates were exothermic, with Δ*H* values of −15 kJ/mol for pNPP, −8 kJ/mol for 5′‐AMP, −6 kJ/mol for 3′‐AMP, and −1 kJ/mol for 4‐MUP (Table S4).

It has been described that class A acid phosphatases are resistant to inhibition by EDTA, tartrate, and phosphate. In agreement with these observations, M2‐32 activity was not inhibited by 1 mM tartrate, 1 mM phosphate, and 10 mM EDTA when assays were carried out at 25 or 50°C. Ishikawa et al. (2000) and Makde et al. (2007) reported that class A 1D2T and 2A96 acid phosphatases could be crystallized when complexed with vanadium. We have found that vanadate is a potent inhibitor of M2‐32 activity, with concentrations of 0.1–1 mM provoking a 90% reduction in activity. In contrast, 1 mM molybdate caused only a 34% decrease in activity. Moreover, incubation of M2‐32 acid phosphatase with 0.1–1 mM of Mg^2+^, Zn^2+^, Cu^2+^, and Mn^2+^ had no significant effect on its activity.

### 2.5 M2‐32 crystal structure and identification of catalytic residues

The truncated model generated by AF2 enabled the determination of the M2‐32 crystal structure at a resolution of 2.2 Å, derived from a crystal belonging to the *P 22*_*1*_ *2*_*1*_ space group. Data collection parameters and refinement statistics, as well as details of the final model, are summarized in Table 2. The structure revealed that M2‐32 forms a tetramer, with a Matthews coefficient of 2.5 and a solvent content of 51.4%. In the crystal, the tetramer adopts a dimer‐of‐dimers arrangement (A‐C/B‐D), with a buried area of 1129.5/1137.1 Å^2^, and a free energy change upon interface formation of −23.5/−22.8 kcal/mol, as determined by the PISA server (Krissinel & Henrick, 2007). This was calculated without considering the six and seven hydrogen bonds formed at the dimer interface, which confirms the homodimer organization of the protein. In each dimer, 21 residues stabilize the structure through 90 non‐bonded contacts, as determined by PDBsum (Laskowski et al., 2018).

**TABLE 2:** Data collection and refinement statistics. Statistics for the highest‐resolution shell are shown in parentheses.

	M2‐32
Data collection	
Synchrotron/beamline	XALOC‐ALBA
Space group	P 2 2_1_ 2_1_
Cell dimensions:	
*a*, *b*, *c* (Å)	61.07, 123.64, 138.65
*α*, *β*, *γ* (°)	90.00, 90.00, 90.00
Unique reflexions	54,038 (4384)
Resolution range	69.32–2.20 (2.26–2.20)
Completeness (%)	99.8 (100.0)
Multiplicity	5.0 (5.3)
Mean I/sigma(I)	5.9 (1.5)
Wilson B‐factor (Å^2^)	35.5
*R*‐merge (%)	15.4 (98.1)
CC1/2 (%)	99.4 (62.9)
Refinement	
No. reflections	53,930 (5301)
*R*_work_/*R*_free_	20.36/24.24
No. atoms	75,080
Protein	7005
Ligand/ion	129
Water	374
Root mean square deviations	
Bond lengths (Å)	0.005
Bond angles (°)	0.67
Ramachandran:	
Favored (%)	98.18
Outliers (%)	0.21
Average B‐factor (Å^2^)	42.59
Macromolecules	42.34
Ligands	56.44
Solvent	42.59
PDB ID	9HTZ

**FIGURE 4:**
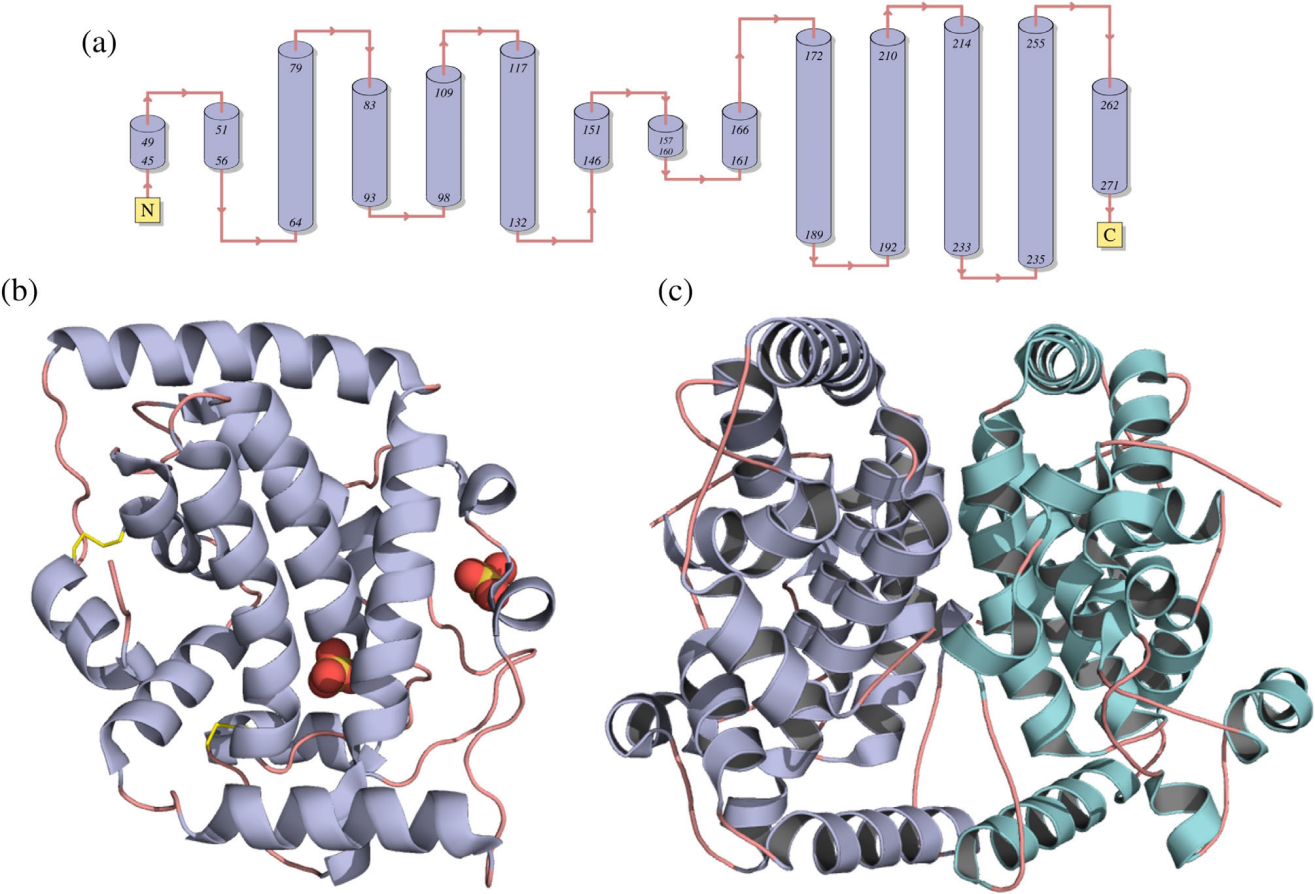
Structural elements of M2‐32. (a) Secondary structural elements of M2‐32 protein showing 14 helices. (b) 3D distribution of the helices in the monomer, including the two disulfide bridges (yellow sticks) and the two sulfates as spheres. (c) The functional dimer of M2‐32.

Each monomer includes residues 45–271 exhibiting an all‐alpha fold arranged with 14 helices (60%), connected by turns (5%) and coils (35%) regions (Figure 4a). The structure is stabilized by two disulfide bridges and belongs to the “acid phosphatase class A” protein fold superfamily (SCOP). The first disulfide bridge forms between Cys105 in helix H4 and Cys263 in helix H14, while the second bridge links Cys156 and Cys210, both residing in the loops regions (Figure 4b).

A structural comparison with the DALI server revealed that M2‐32 shares a high degree of structural similarity with other acid phosphatases (Table S5), including the tethered PhoN from *S. typhimurium* (PDB ID. 2A96) (Makde et al., 2007), the acid phosphatase from *Klebsiella pneumoniae* (strain 342) (PDB ID. 9JQ0, unpublished) or the acid phosphatase from *E. blattae* (PDB IDs. 1IW8) (Ishikawa et al., 2000), with a root mean square deviation (RMSD) of less than 2.0 Å (Figures S3 and S4), despite sequence identity lower than 50% (Table S5). Among the top 10 structural models with the highest *Z*‐scores, only the three previously compared models and the chloroperoxidase from *Streptomyces* sp. CNQ‐525 (PDB ID. 3W36) (McKinnie et al., 2018) also contains at least one disulfide bridge. M2‐32, however, presents an additional extra bridge on the opposite side to the active site of the enzyme. Consistent results were obtained when examining the dimer interface similar for structural similarity using PISA. The top five scored structures identified included several PhoN models from *S. typhimurium* with phosphate or tungstate (PDB ID. 2A96 and 2AKC), the T159D mutant (PDB ID. 2IPB) (Makde et al., 2007) and two models of the acid phosphatase from *E. blattae* in complex with molybdate (PDB IDs. 1EOI) (Ishikawa et al., 2000), and its double mutant G74D/I153T in complex with sulfate (PDB IDs. 1IW8), confirming the dimeric functional oligomerization state of M2‐32.

Docking analysis of 5′‐AMP and 3′‐AMP was performed with the crystallographic model using Autodock Vina (Trott & Olson, 2010). The results are shown in Figure S5. Only poses where the phosphate group of the substrates occupied a similar position as the sulfate molecule in M2‐32 (between His174 and His213) were considered. 5′‐AMP and 3′‐AMP presented estimated free energies of −5.25 and −5.61 kcal/mol, respectively. Residues within 4 Å of 5′‐AMP and 3′‐AMP ligands include K34, L95, G132, G133, S134, Y136, R146, L163, L164, D167, S169, P171, S172, G173, H174, S175, R207, A212, and H213 (the residues at the binding distance of the sulfate molecule in the M2‐32 crystallographic structure are underlined).

**FIGURE 5:**
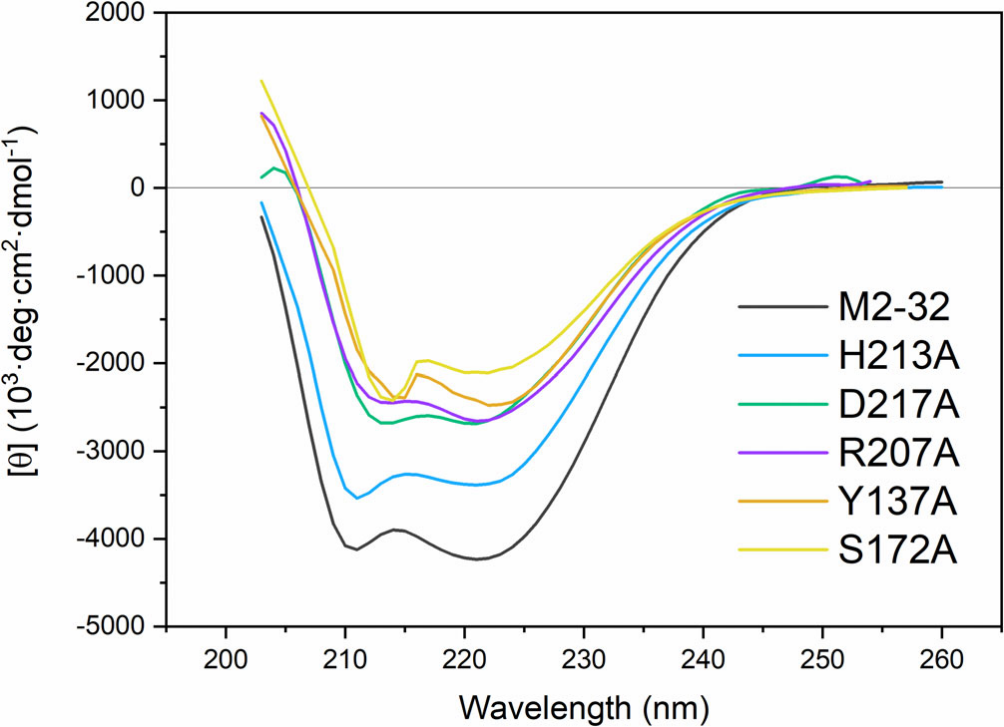
Secondary structure of M2‐32 protein and its mutant variants. Far‐UV circular dichroism (CD) spectra of the M2‐32 protein and its mutants in catalytic residues and substrate binding domain. Spectra were recorded at 25°C in phosphate buffer (10 mM, pH 7.4) with a protein concentration of 18–20 μM. The CD signal is normalized as molar ellipticity units.

Based on the findings of Ishikawa et al. (2000), the residues His174, Arg207, His213, and Asp217 in M2‐32 correspond to the catalytic residues identified in 1D2T. To investigate their role in catalysis, we generated mutants by site‐directed mutagenesis, substituting each residue by alanine. The mutants M2‐32(R207A), M2‐32(H174A), M2‐32(H213A), and M2‐32(D217A) were cloned in the pET28(b) vector and expressed in *E. coli*. The proteins were purified to homogeneity following the same protocol as for the wild‐type protein. Enzymatic assays revealed that all mutants exhibited a >99% reduction in catalytic activity against pNPP, confirming the essential role of these residues in catalysis (Figure S6). To assess whether these mutations affected secondary structure or overall folding, circular dichroism (CD) spectra were recorded for the wild‐type M2‐32 protein and the H213A, H174A, D217A, and R207A mutants. Spectra were collected at 25°C in phosphate buffer (10 mM, pH 7.4), with protein concentrations of ~20 μM. As expected, the M2‐32 protein displayed characteristic α‐helical minima at 208 and 222 nm. All mutants exhibited moderate reductions in ellipticity, consistent with retention of secondary structure and a limited loss of α‐helical content. These minor spectral changes suggest that mutations at catalytic residues have a limited effect on the overall protein fold (Figure 5).

Based on the molecular docking results, we further mutated Tyr136 and Ser172 residues in the environment of the catalytic pocket and generated alanine mutants at these two positions, cloned and expressed the mutant variants. While the Ser172 mutant completely lost activity (>99%), replacing Tyr136 with alanine resulted in a statistically significant reduction in activity but still retained about 10% activity, underscoring their critical role in substrate recognition (Figure S5). Kinetic analysis indicates that the decrease in activity of Y36A was linked to a decrease in the rate of catalysis rather than in substrate affinity.

## 3 DISCUSSION

The in silico analysis of bacterial acid phosphatases identified three distinct groups at the sequence level, each group being characterized by a specific profile as defined by Udaondo et al. (Udaondo et al., 2020). Multi‐alignment of protein sequences within each class revealed conservation across the entire protein sequence, indicating that active site and key structural elements are embedded within the sequence, rather than confined to specific domains.

Notably, regardless of the acid phosphatase class, different quaternary structures have been described. For example, the quaternary structure of the class B AphA acid phosphatase consists of four identical 25 kDa subunits that form a flat molecule with two catalytic sites on each face (Felts et al., 2006). Each catalytic site contains a Mg^2+^ ion (or other metal ion), which interacts with three of the four conserved aspartate residues typical of the catalytic site of the DDDD superfamily. Similarly, class C acid phosphatases feature four catalytic aspartic residues, arranged in bipartite motifs with the consensus sequence X‐D‐I/L‐D‐E‐T and X‐X‐GD(N/T)‐L‐X‐D‐F. Class C proteins, typically 25–30 kDa in size, adopt varied quaternary structures; e.g., a monomer in *Clostridium*, a trimer in *Chryseobacterium*, and a decamer in FS6 enzyme rescued from a soil metagenomics library (Felts et al., 2006; Passariello et al., 2003; Recio et al., 2024; Reilly et al., 2009; Reilly & Calcutt, 2004). Class A acid phosphatases sequence alignment (Figure S1) and structural studies highlight a homodimeric core, where each subunit holds its catalytic site. Class A acid phosphatases are categorized as histidine phosphatases and contain a conserved sequence motif, KX_6_RP‐(X_12–54_)‐PSGH‐(X_31–54_)‐SRX_5_HX_3_D, identified by that which is involved in catalysis (Stukey & Carman, 1997). The crystal structure of the class A 1D2T from *E. blattae* was previously resolved at 1.9 Å and revealed a homohexamer, organized as a trimer of dimers (Ishikawa et al., 2000). Similarly, the 2A96 acid phosphatase of *Salmonella* crystallizes as a tetramer. Interestingly, our size exclusion chromatography analysis and equilibrium sedimentation analyses revealed that M2‐32 is a functional dimer in solution, with over 97% of the protein present in the dimeric state (see Figures S2 and Table S2). However, the M2‐32 protein crystallized as a tetramer, specifically a dimer‐of‐dimers, with a structure resolved at 2.2 Å.

It is well established that some dimeric proteins, while existing as dimers in solution, can adopt tetrameric conformations upon crystallization (Au et al., 1999; Berneburg et al., 2022; Fraser et al., 2016; Pazy et al., 2003; Srivastava et al., 2017; Xu et al., 1990). This shift is often driven by specific interactions (hydrogen bonds, electrostatic forces, and van der Waals contacts) within the crystal lattice (Luo et al., 2015). During crystallization, protein molecules pack in a highly ordered manner, and these lattice‐driven interactions can occasionally favor a higher‐order oligomeric state (Fraser et al., 2016; Luo et al., 2015). Several studies have reported this phenomenon, in which functional dimers in solution crystallize as tetramers; notable examples include streptavidin A (Pazy et al., 2003), and glucose‐6‐phosphate dehydrogenase (Au et al., 1999; Berneburg et al., 2022).

With regards to M2‐32, the monomer exhibits a typical all‐alpha‐helical fold, with nearly 60% of its amino acid residues forming 14 alpha‐helices (Figure 4a). This is consistent with our CD data, and the monomer fold closely resembles that of 1D2T with an RMSD difference of 1.3 Å for the C^alpha^ atoms (Figures S3 and S4).

Acid phosphatases of the three classes, that is, the AphA (class B), class C acid phosphatases (*Clostridium perfrigens*, *Chryobacterium*, FS6) (Recio et al., 2024; Reilly & Calcutt, 2004) and class A 1D2T, exhibit optimal activity at slightly acidic pH, that is, around 5.5. While M2‐32 exhibits a substrate profile similar to other bacterial acid phosphatases (Pond et al., 1989), it is distinct in that it remains active in a broad range of pH (from 4.0 to 8.5), utilizing both archetypal substrates (e.g., *p*‐nitrophenylphosphate) and physiological substrates (i.e., 5′‐ and 3′‐mononucleotides). Given that M2‐32 was rescued from a metagenomic library and its profile of substrates includes only nucleotides, we speculate that M2‐32 may function in its native host to scavenge mononucleotides, thereby providing cells with inorganic phosphate and nucleosides. The broad pH activity profile of M2‐32 observed with both artificial and physiological substrates is unusual. A similar observation has been reported for the extracellular class C *Staphylococcus aureus* acid phosphatase, although data on physiological substrates are not available (Du Plessis et al., 2002). It is worth noting that 2A96 from *Salmonella* exhibits peak activity at pH 5.5; however, a distal mutant (L39T) relative to the catalytic site displayed activity from pH 4 to 7, albeit at 50% of the *V*_max_ (Makde et al., 2007). The underlying causes of decreased activity at lower pH values remain unclear but might be linked to changes in the overall ionization state of the protein.

DSF assays suggest that M2‐32 is a thermotolerant enzyme with an unfolding *T*_m_ of 47°C and, accordingly, this high stability may be conferred by the presence of two sulfide bridges in the monomer, which contrasts with most acid phosphatases that have only one disulfide bridge (Table S5). M2‐32 exhibited maximal activity with pNPP and nucleotides at 45°C. Regardless of temperature, it should be noted that M2‐32 showed higher efficiency with the physiological substrates (e. g., 5′‐AMP and 3′‐AMP) (*k*_cat_ values in the range between 13 and 103 s^−1^, see Table 1) than with pNPP (0.30–0.45 s^−1^), which is 100‐fold higher than with the archetypal substrate. This preference of enzymes for physiological substrates was also observed for the FS6 acid phosphatase (Recio et al., 2024) and various fungal hydrolases (Daddaoua et al., 2023).

In line with other class A acid phosphatases, M2‐32 is resistant to inhibition by EDTA, suggesting that metals are not involved in catalysis, while Mo and V are inhibitors. The mechanism of catalysis of histidine phosphatases involves a nucleophilic attack by a conserved histidine (residue His 174 in M2‐32) to generate a covalent phosphoenzyme intermediate (Ishikawa et al., 2000), followed by hydrolysis to release inorganic phosphate that requires protonation of the leaving group by another His (residue 213 in M2‐32). In fact, mutating His 174 and His 213 to alanine impaired M2‐32 activity, without significantly affecting its 3D structure as shown by CD assays, confirming their essential roles.

Structural studies of the acid phosphatases 1D2T and A296 in complex with the transition‐state analog molybdate at 2.4 Å validate the mechanism of reaction and provide evidence for the covalent phosphoenzyme state. This agrees with the fact that M2‐32 activity was inhibited by molybdate and vanadate. Docking assays identified Ser 172 and Tyr136 as potential residues involved in catalysis, which was supported by the fact that mutating these residues to alanine significantly reduced (>90%) M2‐32 activity.

## 4 CONCLUSIONS

M2‐32 is a class A acid phosphatase that exhibits broad substrate specificity and robust activity across a wide pH range at temperatures of up to 50°C. The protein refolds spontaneously upon thermal denaturation at temperatures as high as 80°C. The catalytic activity of the enzyme, conserved structural motifs, resistance to EDTA, and inhibition by molybdate and vanadate confirm that M2‐32 is a histidine phosphatase. Comparative sequence and crystallographic analyses within class A acid phosphatase revealed that the core structure is a homodimer, which crystallizes as a tetramer. These findings underscore the diversity in quaternary structures and substrate preferences among bacterial acid phosphatases.

## 5 EXPERIMENTAL PROCEDURES

### 5.1 Bacterial strains, plasmids, and culture media

*E. coli* BL21 (DE3) was utilized for protein overexpression (Hanahan, 1983), while *E. coli* DH5α (Studier et al., 1990) was used for cloning purposes. Bacterial cultures were grown in LB medium at temperatures ranging from 18 to 37°C. The plasmids used or constructed in this study are listed in Table S1. When required, kanamycin (Km) at 25 μg/mL or ampicillin (Ap) at 100 μg/mL was added to the culture medium.

### 5.2 Protein purification

For the protein purification, 1–2 g of cells were resuspended in 25 mL of buffer HAM (40 mM 2‐(N‐morpholino)ethanesulfonic acid (MES), 40 mM acetic acid, 40 mM HEPES, pH 7.0 supplemented with 500 mM NaCl, 1 mM dithiothreitol (DTT), 10% (v/v) glycerol (buffer A), and EDTA‐free protease inhibitor cocktail. Cell lysis was achieved by passing the suspension through a French Press three times at 1000 psi. The lysate was then centrifuged at 6000 × *g* for 30 min, and the resulting supernatant was filtered through sterile 0.22 μm filters. The filtrate was loaded onto a 5 mL His‐Trap chelating column (GE Healthcare, St. Gibes, UK). Proteins were eluted using a gradient of 0–500 mM imidazole in buffer B (buffer A but with 500 mM imidazole). The purity of the protein fractions was analyzed using 12% (w/v) SDS‐PAGE gels. Fractions containing homogeneous proteins were dialyzed overnight in HAM buffer with 150 mM NaCl, adjusting pH to 4, 5.5, 7, and 8.5.

### 5.3 Standard enzymatic activity assays

Enzymatic activity was routinely measured using pNPP as the substrate, following the method described by Recio et al. (2024). All the assays were done in triplicate and repeated at least three times. The standard assay contained 100 μL of 1 μM of protein and 20 μL of 0.115M of pNPP. After 15 min of incubation, 100 μL of 0.5M NaOH was added to stop the reaction, and 800 μL of distilled water was added to bring the total volume to 1.02 mL. Activity assays were run at 25 and 50°C. The absorbance of the reaction product (*ρ*‐nitrophenol) was measured spectrophotometrically at 405 nm using a Tecan Sunrise plate reader (Tecan Austria GmbH). The concentration of *p*‐nitrophenol was calculated using an extinction coefficient at 405 nm of Ʃ = 18,000/M cm. When indicated, inhibitors such as EDTA, tartrate, and phosphate were tested at concentrations of 0.1 and 1 mM.

To evaluate the influence of temperature and pH on the activity, standard assays were carried out over a range of temperatures (20, 30, 40, 50, 60, 70, and 80°C) and pHs (3.5, 4.5, 5.5, 6.5, 7.5, and 8.5). Protein stability was assessed by incubating 1 μM M2‐32 at pH 5.5 for 30 or 60 min at temperatures ranging from 20 to 80°C. The samples were then cooled to 25°C, and the enzymatic activity was determined after the addition of the substrate.

For kinetics analysis, increasing concentrations of pNPP, 5′‐AMP, 3′‐AMP, and 4‐MUP ranging from 0 to 2.102 mM were used. The initial reaction rate (*V*_0_) was plotted as a function of the substrate concentration. The values of *V*_max_ and *K*_m_ were derived from this plot using non‐linear curve fitting with the Michaelis–Menten equation and the Lineweaver‐Burk plot (*R* ^2^ >0.9). The turnover number (*K*_cat_) was calculated from *V*_max_ using the equation *V*_max_/*E*, where *E* is the enzyme concentration.

### 5.4 Determination of the enthalpy of the hydrolysis reaction

Microcalorimetric assays were carried out at 25°C using a VP‐microcalorimeter (Microcal, Amherst, Massachusetts, USA) (Krell, 2008; Todd & Gomez, 2001). Purified phosphatase and substrate solutions underwent dialysis against HAM supplemented with 150 mM NaCl, 1 mM DTT, and 10% [v/v] glycerol at pH 5.5.

During titration, 20–30 μL of 0.5–1 mM substrate solutions were injected into the assay mixture containing 2.5–5 μM of purified M2‐32 acid phosphatase. Control experiments were performed by injecting the substrate solutions into the buffer without the enzyme. Raw titration data were adjusted for concentration and dilution effects, and data analysis was performed using the “Enzyme kinetics—single injection model” provided by the MicroCal PEAQ‐ITC analysis software. The parameter Δ*H* (reaction enthalpy) was obtained through curve fitting, following the methodology of Abadou and Ladbury (Ababou & Ladbury, 2006).

### 5.5 Analytical ultracentrifugation

An Optima XL‐I analytical ultracentrifuge (Beckman‐Coulter, Palo Alto, CA) equipped with a UV–visible absorbance detection system was used. Sedimentation velocity experiments were conducted at 20°C with an AnTi50 rotor, and absorbance scans were recorded at 280 nm. Samples (at a concentration of 1 and 3 mg/mL) were loaded into epon‐charcoal standard double‐sector centerpieces (12‐mm optical path) and spun at 48,000 rpm. Sedimentation coefficient distributions were analyzed using SEDFIT software (Brown & Schuck, 2008; Zhao et al., 2010). SEDNTERP software (Philo, 2023) was used to correct S values to standard conditions (20°C and water). The apparent weight‐average buoyant molecular weights were calculated by fitting single‐species models to the experimental data using MATLAB. The corresponding protein molecular weights were calculated using a protein partial specific volume of 0.725 cm^3^/g.

### 5.6 Differential scanning fluorimetry

Thermal denaturation assays were conducted using a MyIQ2 Real‐Time PCR instrument (Fernández et al., 2019). Twenty‐five microliters of a preparation containing 10 μM of M2‐32 in HAM buffer pH 5.5 supplemented with SYPRO orange (Life Technologies) at a concentration of 5× was heated from 23 to 85°C at a scan rate of 1°C/min. The protein unfolding was monitored by detecting changes in SYPRO fluorescence. The unfolding temperature (*T*_m_) was calculated using Bio‐Rad iQ5 software using the first derivative value from the raw fluorescence data. Protein refolding assays were carried out by heating samples at 80°C for 1 h, cooling them at 25°C, and then determining *T*_m_ post‐refolding. Up to four denaturation/refolding cycles were evaluated.

### 5.7 Size exclusion chromatography

To determine the oligomeric state of M2‐32 phosphatase in solution, we used analytical gel filtration chromatography using an Åkta FLPC system (Cytiva). Purified protein (30 μM) was loaded onto a HiPrep 26/60 Sephacryl S 500HR column (Cytiva) equilibrated in buffer C (40 mM HEPES‐acetic acid‐MES, 150 mM NaCl and 10% (v/v) Glycerol, pH 5.5). M2‐32 was eluted at a constant flow rate of 1 mL/min, and the absorbance of the eluate was monitored at 280 nm. The molecular mass of M2‐32 was estimated from a plot of the elution volume against the Ln of the molecular weight of standard calibration proteins, namely, albumin from chicken egg white (45 kDa), carbonic anhydrase from bovine erythrocytes (29 kDa), α‐lactoalbumin from bovine milk (14.2 kDa) and albumin from bovine serum (66 and 132 kDa) (Sigma) (Figure S2).

### 5.8 Substrate profile of M2‐32

The substrate profile of M2‐32 was evaluated by measuring phosphate release from a diverse range of organophosphorous compounds, including nucleotides, sugar phosphates, and amino acid phosphates (see Table S3). The assays were done in HAM buffer at four different pHs: 4, 5.5, 7, and 8.5. Orthophosphate released was quantified using the “Malachite Green Phosphate Assay” kit (Sigma‐Aldrich) with absorbance measured at 660 nm using a TECAN Sunrise multiwell plate reader. A reaction was considered positive for M2‐32 when the phosphate released exceeded 10 μM. This represents the lower detection limit for phosphate release under our experimental conditions (Green Malachite, SIGMA catalog number MAK 307). The assays were repeated at least three times.

### 5.9 Plasmid DNA extraction and generation of mutant proteins M2‐32 in residues involved in substrate binding and hydrolysis

The NZYMiniprep commercial kit (NZYTech) was used according to the manufacturer's instructions to obtain highly pure plasmid DNA. Cells from a 24‐h culture in LB medium (2 mL) supplemented with the appropriate antibiotic were harvested by centrifugation at 13,000 rpm for 1 min.

To generate mutations the pET28(b)::M2‐32 plasmid served as a template. Mutants were generated using the method described by Li and Wilkinson (1997) with modifications. It is based on preparing two PCR mixes for each mutant. The PCR Mix 1 reaction contained 100 ng of template plasmid DNA, 150 mM of each dNTP, 1 U Pfu turbo DNA Polymerase (Stratagene), 1 U Taq polymerase (Roche), 200 ng of primer M2‐32 Fw, and 1× buffer supplied with pfu turbo enzyme, in a total volume of 24 μL. The forward primers used for each mutant were: M2‐32 Fw2 for M2‐32 (R207A), M2‐32 Fw3 for M2‐32 (H213A), and M2‐32 Fw 4 for M2‐32 (D217A). The PCR Mix 2 was as above, but containing 200 ng of M2‐32 Reverse primers (see Table S6). The reverse primers used for each mutant were: M2‐32 Rv 2 for M2‐32 (R207A), M2‐32 Rv 3 for M2‐32 (H213A), and M2‐32 Rv 4 for M2‐32 (D217A). The triplet nucleotides encoding the substituted amino acid are underlined in Table S6.

The thermocycler program for Mix 1 and Mix 2 separately was three cycles at 95°C—30 s, 55°C—60 s, and 68°C—6 min dropping to 14°C—10 min. DNA was denatured at 95°C for 2 min before the first cycle. Once the Mix 1 and Mix 2 reactions were tempered, both mixtures were pooled in a single 0.2 mL Eppendorf tube, and a 22‐cycle thermal cycler program at 95°C—30 s, 55°C—60 s, and 68°C—6 min dropping to 14°C—10 min was set up. It should be noted that the DNA was denatured at 95°C for 2 min before the first cycle. Upon amplification, then 15 μL of the product of this reaction was cleaved with 10 U of *Dpn*I (New England Biolabs) for 14 h at 37°C. Subsequently, the entire reaction was transformed into competent *E. coli* DH5α cells by heat shock and spread on solid Luria Bertani medium supplemented with 25 μg/mL Km. The identity of the mutants was confirmed by sequencing the mutant allele with the T7 and T7T primers. Then the different plasmid DNA alleles were digested with *Nde*I and *EcoR*I and subcloned into pET28(b).

Mutants in which residues His174, Ser 172, and Y136 were replaced by alanine were synthesized in vitro by GeneScript. The bona fide nature of the mutants was confirmed by Sanger sequencing.

All mutant variants were cloned in pET28(b) vector and expressed in *E. coli* BL21 (DE3), following the same procedure as for the wild‐type protein. SDS‐PAGE analysis revealed that the expression levels of the mutant proteins were similar to those of the parental protein, with final concentrations between 0.5 and 1.2 mg/mL.

### 5.10 Circular dichroism

CD in a Jasco J‐715 spectropolarimeter (Jasco, Tokyo, Japan) spectra (260–200 nm) was made in a 1 mm path‐length quartz cuvette at a protein concentration of ~20 μM in  10 mM phosphate buffer pH 7.0. Spectra were averaged from five scans recorded at a rate of 100 nm/min, 1 nm step resolution, 1 s response, and 1 nm bandwidth.

### 5.11 Docking assays

Docking experiments with M2‐32 were performed using Autodock Vina (Eberhardt et al., 2021; González‐Ramírez et al., 2017; Trott & Olson, 2010) on the Galaxy server (https://usegalaxy.eu). Automated restrained docking calculations were carried out (exhaustiveness = 8, grid size of *X* = *Y* = *Z* = 10). Automated preparation of the receptor file (exclusion of water molecules and hydrogen atoms addition) was carried out using the MGLTools programming package implemented in the Galaxy Server. No flexible chains were used for the simulations, and a pH value of 7.4 was set up using the Vina scoring function. Only those poses in which the phosphate group of the substrate occupied a position analogous to that of the sulfate molecule in M2‐32 (situated between His174 and His213) were considered.

### 5.12 Protein crystallization and data collection

Freshly purified protein in 40 mM HEPES, 40 mM sodium acetate, and 40 mM MES containing 50 mM NaCl and 5% glycerol (v/v) was concentrated to approximately 9 mg/mL and used for initial crystallization screenings. Screenings were conducted with Hampton Research Crystal Screens I and II using the hanging‐drop vapor diffusion technique. Crystals were grown at 20°C by mixing equal volumes (1 μL each) of protein solution and reservoir solution.

Well‐faceted crystals suitable for x‐ray analysis were identified under a polarized light microscope. The selected M2‐32 crystals were transferred to a drop of mother liquor solution supplemented with 15% (v/v) glycerol as a cryo‐protectant, then immediately flash‐cooled in liquid nitrogen for storage until data collection. The crystals were tested at ID30B (ESRF, Grenoble, France) and XALOC (ALBA, Barcelona, Spain) beamlines, with the final x‐ray diffraction data collected at XALOC. Diffraction frames were indexed and integrated with XDS (González‐Ramírez et al., 2017), and subsequently reduced and merged with Aimless (Evans & Murshudov, 2013). The structural model was determined by the molecular replacement method using Molrep (Vagin & Teplyakov, 2010) from the CCP4 suite (Project, 1994), employing the AF2 (Jumper et al., 2021) computed model. Refinement was carried out with Phenix.refine (Mustyakimov et al., 2010), followed by additional rounds in REFMAC (Murshudov et al., 2011) from the Phenix (Adams et al., 2010) and CCP4 (Painter & Merritt, 2006) suites, and finalized through several cycles of refinement applying Translation/Libration/Screw (TLS) parameterization (Painter & Merritt, 2006). Model inspection and manual corrections were done with COOT (Emsley et al., 2010). Model quality was monitored using Molprobity (Chen et al., 2010) integrated within the PHENIX package and PDB‐REDO (Adams et al., 2010). Data collection and refinement statistics are summarized in Table 2.

## AUTHOR CONTRIBUTIONS

**Maria‐Isabel Recio:** Writing – review and editing; methodology; validation; investigation; writing – original draft. **José A. Gavira:** Investigation; data curation; visualization; supervision; writing – original draft; writing – review and editing. **Jesús de La Torre:** Investigation; methodology; writing – review and editing. **Mario Cano‐Muñoz:** Investigation; methodology; writing – review and editing. **Sergio Martínez‐Rodriguez:** Investigation; writing – review and editing; visualization; methodology. **Abdelali Daddaoua:** Investigation; writing – review and editing; visualization; methodology. **Estrella Duque:** Investigation; supervision; writing – original draft; writing – review and editing. **Juan L. Ramos:** Conceptualization; writing – original draft; writing – review and editing; supervision; project administration; funding acquisition; methodology.

## FUNDING INFORMATION

The work in our laboratory was funded by grant PDI‐2021‐123469OB‐100 funded by MICIU/AEI/501100011033 and ERDF a way making Europe and Granat P20‐00049 by the Regional Goverment of Andalusia

## CONFLICT OF INTEREST STATEMENT

The authors declare no conflict of interest.

## Supporting information


**Table S1.** Strains and plasmids used in this study.
**Table S2**. Parameters of the ultracentrifugation sedimentation analysis.
**Table S3**. Organophosphorus compounds tested in this study.
**Table S4**. Enthalpy variations obtained by ITC after enzyme and substrate binding.
**Table S5**. Structural alignment of the M2‐32 with structures deposited in the Protein Data Bank.
**Table S6**. Oligonucleotide primers used in this study.

**Figure S1.** Sequence alignment of the proteins described as NSAP with M2‐32. Created with CLC Sequence Viewer. Conservation of the sequence is showed by color scale (0% blue – 100% intense red).

**Figure S2.** Oligomeric state of M2‐32 phosphatase in solution. Purified protein (30 μM) was loaded onto a HiPrep 26/60 Sephacryl S 500HR column (Cytiva) equilibrated in 40 mM HEPES‐Acetic acid‐MES, 150 mM NaCl and 10% (v/v) glycerol, buffer at pH 5.5, in an Åkta FLPC system (Cytiva). M2‐32 was eluted at a constant flow rate of 1 mL/min, and the absorbance of the eluate was monitored at 280 nm. The molecular mass of M2‐32 was estimated from a plot of the elution volume against the Ln of the molecular weight of standard calibration proteins from Sigma, namely: (A) albumin from bovine serum (dimer, 132 kDa), (B) albumin from bovine serum (monomer, 66 kDa); (C) albumin from chicken egg white (45 kDa); (D) carbonic anhydrase from bovine erythrocytes (29 kDa), and (E) α‐lactoalbumin from bovine milk (14.2 kDa).

**Figure S3.** Ribbons representation of the superimposition of M2‐32 (red) and the acid phosphatase from *Escherichia blattae* (PDB ID 1IW8, cyan).

**Figure S4.** Ribbons representation of the superimposition of M2‐32 (red) and the PhoN protein of *Salmonella typhimurium* (PDB IDs 2A96, green), the acid phosphatase from *Escherichia blattae* (PDB IDs. 1IW8, blue) and the acid phosphatase from *Klebsiella pneumonia* (strain 342) (PDB ID 9JQ0, yellow).

**Figure S5.** Docking of 5′‐AMP and 3′‐AMP within a 1.0 nm box centered at the sulfate moiety obtained with Autodock Vina.

**Figure S6.** Activity of M2‐32 mutants and wild‐type. Values are the average of three different replicates done in triplicate. Statistical analysis were carried out with ANOVA, *p < 0.05.

## Data Availability

The data that supports the findings of this study are available in the supplementary material of this article.
