# Supplementary material for: Thermotolerant class A acid phosphatase active across broad pH range and diverse substrates
Source: Protein Sci. 2025 Aug 15;34(9):e70244. doi: 10.1002/pro.70244 (PMC12356135; doi:10.1002/pro.70244)
Supplement: Supplementary file 1 — Table S1. Strains and plasmids used in this study. Table S2. Parameters of the ultracentrifugation sedimentation analysis. Table S3. Organophosphorus compounds tested in this study. Table S4. Enthalpy variations obtained by ITC after enzyme and substrate binding. Table S5. Structural alignment of the M2‐32 with structures deposited in the Protein Data Bank. Table S6. Oligonucleotide primers used in this study. [file PRO-34-e70244-s006.docx]

**SUPPLEMENTARY MATERIAL**

**Thermotolerant class A acid phosphatase active across broad pH range and diverse substrates**

Maria-Isabel Recio^1^, José A. Gavira^2^, Jesús de La Torre^1^ , Mario Cano-Muñoz^1^ , Sergio Martínez^3^, Abdelali Daddaoua^4^, Estrella Duque^1^, and Juan L. Ramos^1^*

^1^Estación Experimental del Zaidín. Consejo Superior de Investigaciones Científicas.

c/ Profesor Albareda 1, 18008 Granada, Spain

^2^Instituto Andaluz de Ciencias de la Tierra. Consejo Superior de Investigaciones Científicas, c/ Paseo de las Palmeras, 18100 Armilla, Spain

^3^Departamento de Bioquímica y Biología Molecular III e Inmunología, Universidad de Granada, c/ Avenida de la Innovación 11, 18019 Granada, Spain

^4^Departamento de Bioquímica y Biología Molecular II, School of Pharamacy, 18071 Granada, Spain

**LEGENDS FOR SUPPLEMENTARY FIGURES**

**Supplementary Figure 1:** Sequence alignment of the proteins described as NSAP with M2-32. Created with CLC Sequence Viewer. Conservation of the sequence is showed by color scale (0% blue – 100% intense red).

**Supplementary Figure 2:** Oligomeric state of M2-32 phosphatase in solution. Purified protein (30 μM) was loaded onto a HiPrep 26/60 Sephacryl S 500HR column (Cytiva) equilibrated in 40 mM HEPES-Acetic acid-MES, 150 mM NaCl and 10% (v/v) Glycerol, buffer at pH 5.5, in an Åkta FLPC system (Cytiva. M2-32 was eluted at a constant flow rate of 1 ml/min, and the absorbance of the eluate was monitored at 280 nm. The molecular mass of M2-32 was estimated from a plot of the elution volume against the Ln of the molecular weight of standard calibration proteins from Sigma , namely: A, albumin from bovine serum (dimer, 132 kDa), B, albumin from bovine serum ( monomer, 66 kDa).; C: albumin from chicken egg white (45 kDa),; D: carbonic anhydrase from bovine erythrocytes (29 kDa), and E : α-lactoalbumin from bovine milk (14.2 kDa).

**Supplementary Figure 3:** Ribbons representation of the superimposition of M2-32 (red) and the acid phosphatase from *Escherichia blattae* (PDB ID 1IW8, cyan).

**Supplementary Figure 4:** Ribbons representation of the superimposition of M2-32 (red) and the PhoN protein of *Salmonella typhimurium* (PDB IDs 2A96, green), the acid phosphatase from *Escherichia blattae* (PDB IDs. 1IW8, blue) and the acid phosphatase from *Klebsiella pneumonia* (strain 342) (PDB ID 9JQ0, yellow).

**Supplementary Figure 5:** Docking of 5’AMP and 3’AMP within a 1.0 nm box centered at the sulphate moiety obtained with Autodock Vina.

**Supplementary Figure 6:** Activity of M2-32 mutants and wild type. Values are the average of three different replicates done in triplicate. Statistical analysis were carried out with ANOVA, * p< 0.05

**Suppl. Table 1. Strains and plasmids used in this study**

| **Strain** | **Genotype or relevant characteristics** | **Reference** |
| --- | --- | --- |
| *E. coli* DH5α | F^-^, *supE*44, *hsdR*17 (rK^-^rK^+^), *recA*1, *gyrA*96 (Nal^R^), *endA*1, *thi*-1, *relA*1 D(*lacZYA-argF*), (Ø80lacZDM15), U169 | Hanahan et al., 1983 |
| *E. coli* BL21 (DE3) | F-, *ompI*, *hsdS* (r^-^B m^-^B), *gal, dam, met* | Studier et al., 1990 |
| **Plasmid** | **Genotype or relevant characteristics** | **Reference** |
| pET28(b) | Expression vector, 6xHIS, (Km^R^). | Novagen |
| pGEM-T | Vector to clone PCR products. (Ap^R^) | Promega |
| pET28(b)::M2-32 | pET28(b) bearing the ORF M2-32 hypothetical protein. (Km^R^). | This study |
| pGEMT::M2-32(R207A) | pGEMT bearing the ORF M2-32 with the arginine at position 207 replaced by alanine. (Km^R^). | This study |
| pET28(b)::M2-32(R207A) | pET28(b) bearing the ORF M2-32 with the arginine at position 207 replaced by alanine. (Km^R^). | This study |
| pGEMT::M2-32(H213A) | pGEMT bearing the ORF M2-32 with the histidine at position 213 replaced by alanine. (Km^R^). | This study |
| pET28(b)::M2-32(H213A) | pET28(b) bearing the ORF M2-32 with the histidine at position 213 replaced by alanine. (Km^R^). | This study |
| pGEMT::M2-32(D217A) | pGEMT bearing the ORF M2-32 with the aspartic acid at position 217 replaced by alanine. (Km^R^). | This study |
| pET28(b)::M2-32(D217A) | pET28(b) bearing the ORF M2-32 with the aspartic acid at position 217 replaced by alanine. (Km^R^). | This study |

Ap^R^ and Km^R^ stand for resistance to ampicillin and kanamycin, respectively.

**Supplementary Table 2**: Parameters of the ultracentrifugation sedimentation analysis**.**

|  | **M2-32** |
| --- | --- |
| **Peak** | 97.2% |
| **sw (S)** | 2.250 |
| **sw (20,w) (S)** | 3.632 |
| **c** | 0.5745 |
| **% of total** | 93.127 |
| **Coefficient of frictionl (f/f_0_)** | 1.383 |
| **Mw (Da)** | 50354 |
| **vbar** | 0.725 |
| **vbar20** | 0.725 |
| **Hydration** | 0.3 |
| **Buffer density** | 1.03977 |
| **Relative viscosity** | 1.4381 |
| **Minimun Mw for compact sphere (Da)** | 30955 |
| **Stokes Radius (20ºC)** | 3.37 |
| **a/b(oblate)** | 5.0 |
| **a/b(prolate)** | 4.67 |

**Supplementary Table 3.** Organophosphorus compounds tested in this study.

| **Phosphorus source** | **pH 4** | **pH 5.5** | **pH 7** | **pH 8.5** |
| --- | --- | --- | --- | --- |
| *D-3’-phosphoglyceric acid* | - | - | - | - |
| *O-Phospho-L-serine* | - | - | - | - |
| *4-Methylumbelliferyl phosphate* | + | + | + | + |
| *Uridine 5’-monophosphate* | - | - | - | - |
| *Trimethyl phosphate* | - | - | - | - |
| *D-glucose-6-phosphate* | - | - | - | - |
| *D-ribose-5’-phosphate* | - | - | - | - |
| *Adenosine 3’-monophosphate* | + | + | + | + |
| *Adenosine 5’-monophosphate* | + | + | + | + |
| *Carbamyl phosphate* | - | - | - | - |
| *para-nitrophenyl phosphate* | + | + | + | + |

M2-32 at a protein concentration of 1 µM in HAM buffer at the indicted pH was tested with the set of compounds indicated above at a concentration of 1 mM. The phosphate released was determined as indicated in Materials and Methods. (+) means that the phosphorous released from the tested compound was above 10µM.

(-) means that no free phosphate was detected.

**Supplementary Table 4**:

Enthalpy variations obtained by ITC after enzyme and substrate binding. The ITC assays were performed at least three times as described under *Experimental Procedures* section. The ΔH (kJ/mol) values were determined based on the heat released during the completion of substrate hydrolysis. The values given are the average from three independent assays.

|  | **ΔH (kJ/mol)** | | | |
| --- | --- | --- | --- | --- |
|  | **pNPP** | **5’-AMP** | **3’-AMP** | **4-MUP** |
| *pH 5.5* | -15±0 | -8±0.1 | -6±0.8 | -1±0.6 |
|  |  |  |  |  |

**Table S5. Structural alignment of the M2-32 with structures deposited in the Protein Data Bank**.

| **PDB Id.** | **SS-bridges** | **Ligands** | **Bacterial species** | **Z-score** | **rmsd** | **lali** | **nres** | **% id** | **Ref.** |
| --- | --- | --- | --- | --- | --- | --- | --- | --- | --- |
| 2a96 | 1 | PO4 | *Salmonella typhimurium* | 30.2 | 1.7 | 217 | 219 | 42 | [10.1021/bi062180g](https://doi.org/10.1021/bi062180g) |
| 9jq0 | 1 | PO4 | *Klebsiella pneumoniae* | 27.4 | 1.9 | 215 | 232 | 46 | Un-published |
| 1iw8 | 1 | SO4 | *Escherichia blattae* | 26.4 | 1.9 | 209 | 219 | 46 | 10.1093/protein/15.7.539 |
| 7f17 | 0 | - | *Pseudomonas aeruginosa* | 20.9 | 2.4 | 206 | 213 | 26 | ACS Catalysis 11 13397-13407 (2021) |
| 5jki | 0 | WO4 | *Bacillus subtilis* | 12.1 | 3.8 | 166 | 201 | 20 | PubMed id: [**28168443**](http://www.ncbi.nlm.nih.gov/entrez/query.fcgi?cmd=Retrieve&db=PubMed&dopt=Abstract&list_uids=28168443) |
| 6ebu | 0 | SO4 | *Aquifex aeolicus* | 11.9 | 3.4 | 152 | 165 | 16 | Un-published |
| 8cxl | 0 | Mg/Cl | *Streptomyces sp. Cnq-525* | 11.9 | 3.2 | 187 | 472 | 12 | PubMed id: [**35985031**](http://www.ncbi.nlm.nih.gov/entrez/query.fcgi?cmd=Retrieve&db=PubMed&dopt=Abstract&list_uids=35985031) |
| 3w36 | 1 | VO4 | *Streptomyces* | 11.5 | 3.1 | 180 | 471 | 13 | PubMed id: [**35985031**](http://www.ncbi.nlm.nih.gov/entrez/query.fcgi?cmd=Retrieve&db=PubMed&dopt=Abstract&list_uids=35985031) |
| 1idu | 0 | VO4 | *Curvularia inaequalis* | 11.4 | 3.3 | 185 | 576 | 14 | PubMed id: [**9165086**](http://www.ncbi.nlm.nih.gov/entrez/query.fcgi?cmd=Retrieve&db=PubMed&dopt=Abstract&list_uids=9165086) |
| 4usz | 0 | VO4 | Zobellia galactanivorans | 11.4 | 3.5 | 186 | 415 | 16 | DOI: [**10.1128/AEM.02430-14**](http://dx.doi.org/10.1128/AEM.02430-14) |

It is presented the top 10 structures according to the Z-score> than 11.0. Alignments were made with the DALI server (1).

**Supplementary Table 6.** Oligonucleotide primers used in this study.

| **Name** | **5´ 3´ Sequence *** | **Description** |
| --- | --- | --- |
| Reverse 17 | 5´-CAGGAAACAGCTATGAc-3´ | Sequencing of cloned fragments in PCR vectors |
| Universal 17 | 5´-GTTTTCCCAGTCACGAC-3´ | Sequencing of cloned fragments in PCR vectors |
| T7 | 5´-TAATACGACTCACTATAGGG-3´ | Sequencing of cloned fragments in vectors of the  pET system. |
| T7T | 5´-CTAGTTATTGCTCAGCGGT-3´ | Sequencing of cloned fragments in vectors of the  pET system. |
| M2-32 Rv(HindIII) | 5´- CC**AAGCTT**GTGCTGGGTCAGC -3´ | Construction of M2-32 protein. |
| M2-32 Fw(NdeI) | 5´-CGC**CATATG**AAAAAAATACCTGAACCC-3´ | Construction of M2-32 protein. |
| M2-32 Fw2 | 5´- GCATACGGCCAGAGCGCAGTCATCTGCGACGCCCACTG -3´ | Construction of M2-32 (R207A) mutant. |
| M2-32 Rv2 | 5´- GGCGTCGCAGATGACTGCGCTCTGGCCGTATGCCAGGCC -3´ | Construction of M2-32 (R207A) mutant. |
| M2-32 Rv3 | 5´- CACGTCGCTCTGCCATGCGGCGTCGCAGATGACCCGG -3´ | Construction of M2-32 (H213A) mutant. |
| M2-32 Fw3 | 5´- GTCATCTGCGACGCCGCATGGCAGAGCGACGTGGAC - 3´ | Construction of M2-32 (H213A) mutant. |
| M2-32 Fw4 | 5´- GCAGAGCGCAGTGGACGCT - 3´ | Construction of M2-32 (D217A) mutant. |
| M2-32 Rv4 | 5´- AGCGTCCACTGCGCTCTGC - 3´ | Construction of M2-32 (D217A) mutant. |

* Underlined are the nucleotide triplets modified to generate the mutation in the amino acids of the M2-32 proteins. In bold, endonuclease recognition sequences.
