# Supplementary figures and images for: Thermotolerant class A acid phosphatase active across broad pH range and diverse substrates
Source: Protein Sci. 2025 Aug 15;34(9):e70244. doi: 10.1002/pro.70244 (PMC12356135; doi:10.1002/pro.70244)

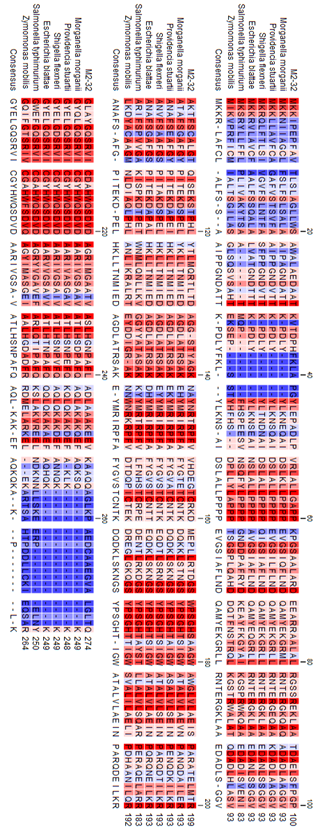

Supplement: Supplementary file 2 — Figure S1. Sequence alignment of the proteins described as NSAP with M2‐32. Created with CLC Sequence Viewer. Conservation of the sequence is showed by color scale (0% blue – 100% intense red). [file PRO-34-e70244-s002.tif]

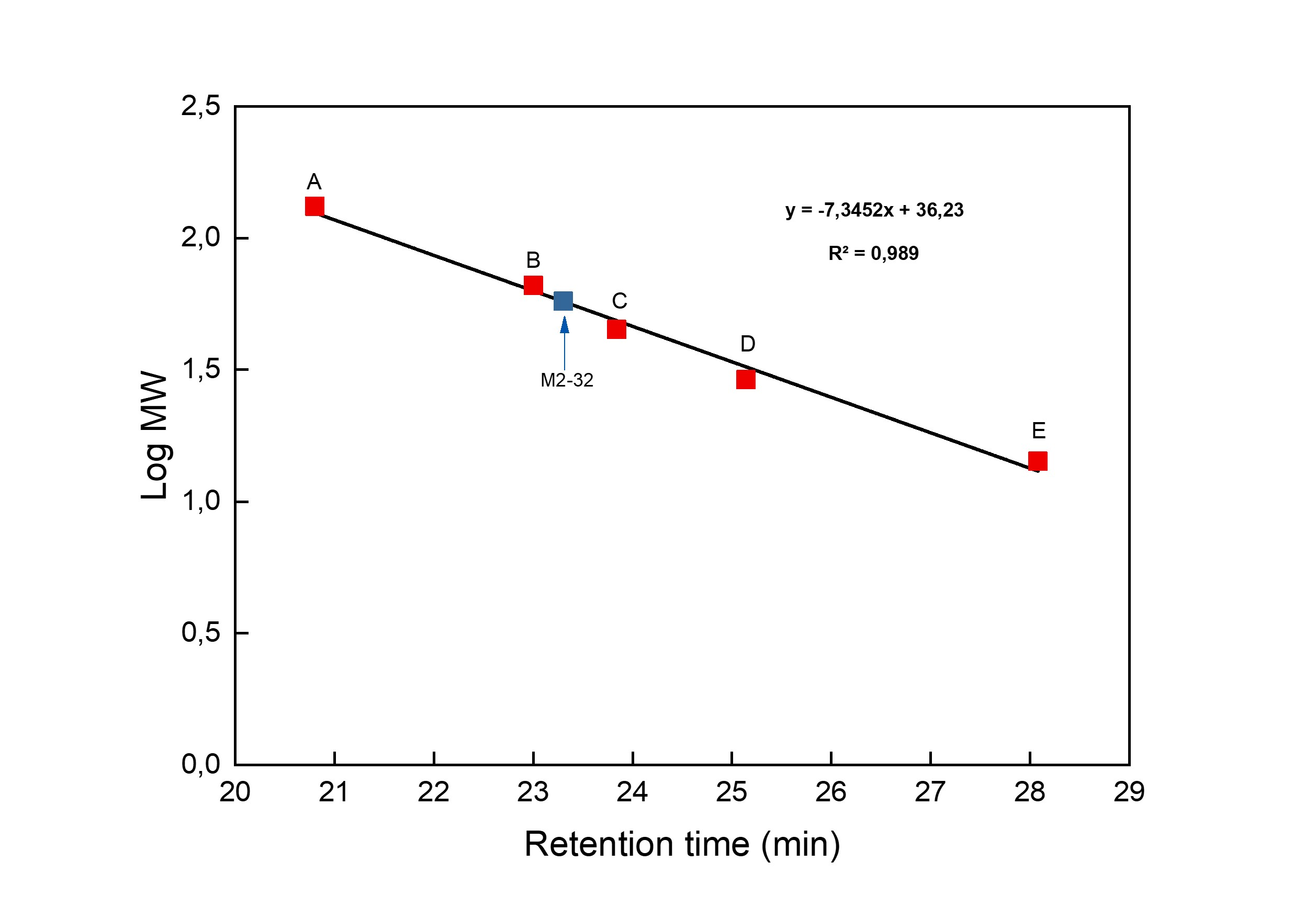

Supplement: Supplementary file 3 — Figure S2. Oligomeric state of M2‐32 phosphatase in solution. Purified protein (30 μM) was loaded onto a HiPrep 26/60 Sephacryl S 500HR column (Cytiva) equilibrated in 40 mM HEPES‐Acetic acid‐MES, 150 mM NaCl and 10% (v/v) glycerol, buffer at pH 5.5, in an Åkta FLPC system (Cytiva). M2‐32 was eluted at a constant flow rate of 1 mL/min, and the absorbance of the eluate was monitored at 280 nm. The molecular mass of M2‐32 was estimated from a plot of the elution volume against the Ln of the molecular weight of standard calibration proteins from Sigma, namely: (A) albumin from bovine serum (dimer, 132 kDa), (B) albumin from bovine serum (monomer, 66 kDa); (C) albumin from chicken egg white (45 kDa); (D) carbonic anhydrase from bovine erythrocytes (29 kDa), and (E) α‐lactoalbumin from bovine milk (14.2 kDa). [file PRO-34-e70244-s004.tif]

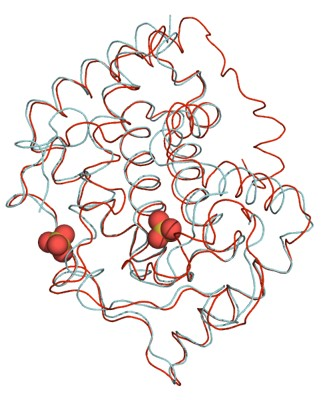

Supplement: Supplementary file 4 — Figure S3. Ribbons representation of the superimposition of M2‐32 (red) and the acid phosphatase from Escherichia blattae (PDB ID 1IW8, cyan). [file PRO-34-e70244-s007.tif]

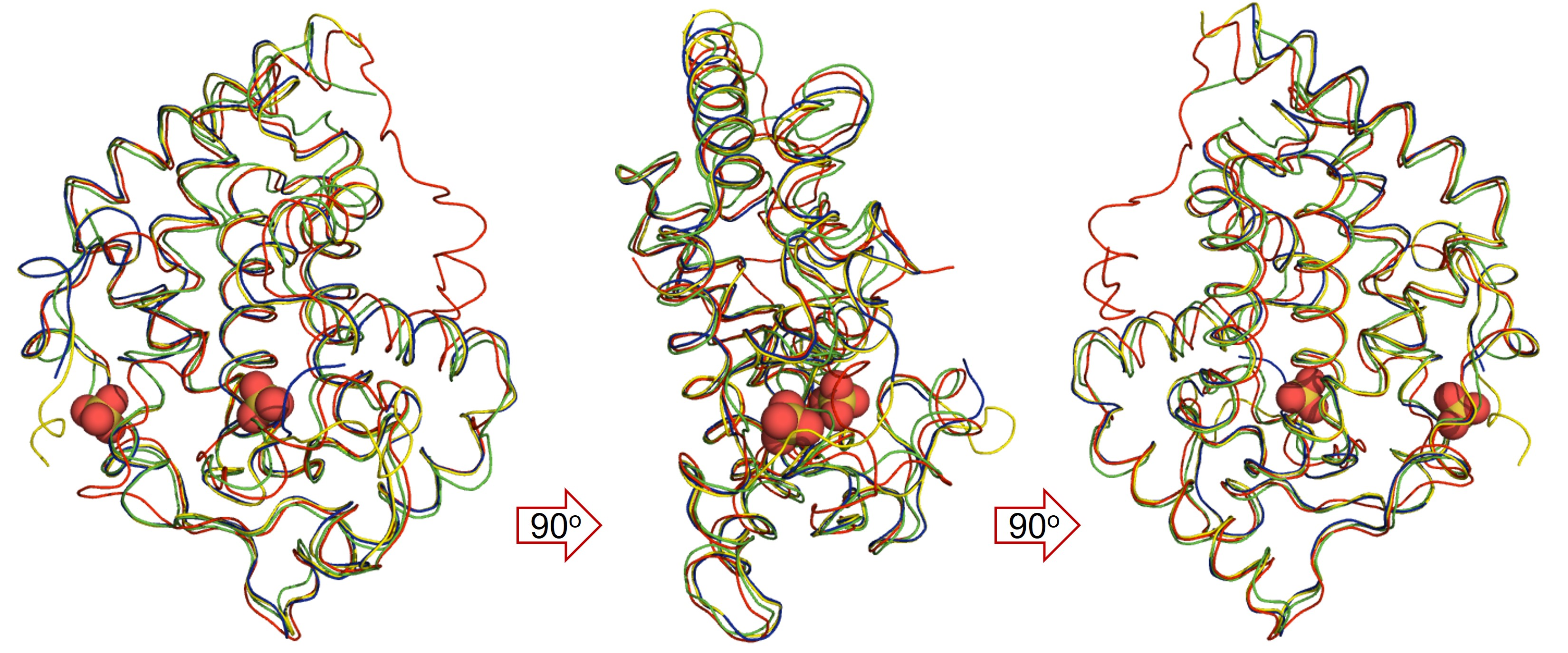

Supplement: Supplementary file 5 — Figure S4. Ribbons representation of the superimposition of M2‐32 (red) and the PhoN protein of Salmonella typhimurium (PDB IDs 2A96, green), the acid phosphatase from Escherichia blattae (PDB IDs. 1IW8, blue) and the acid phosphatase from Klebsiella pneumonia (strain 342) (PDB ID 9JQ0, yellow). [file PRO-34-e70244-s001.tif]

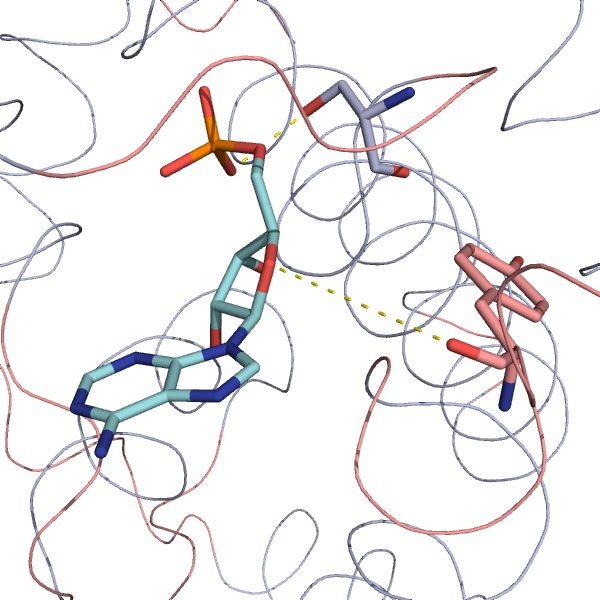

Supplement: Supplementary file 6 — Figure S5. Docking of 5′‐AMP and 3′‐AMP within a 1.0 nm box centered at the sulfate moiety obtained with Autodock Vina. [file PRO-34-e70244-s005.tif]

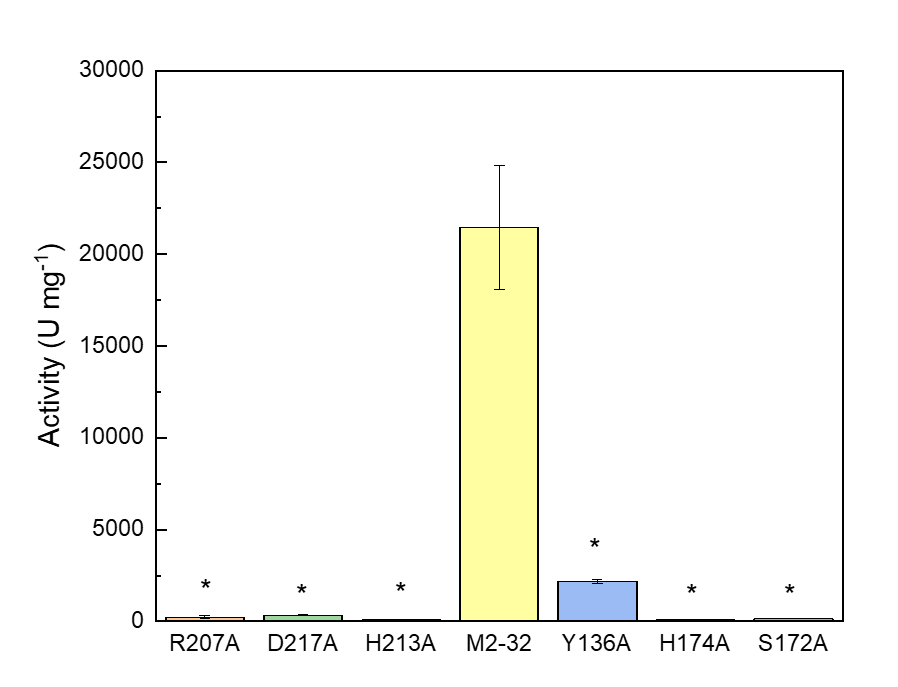

Supplement: Supplementary file 7 — Figure S6. Activity of M2‐32 mutants and wild‐type. Values are the average of three different replicates done in triplicate. Statistical analysis were carried out with ANOVA, *p < 0.05. [file PRO-34-e70244-s003.tif]
